# E3 Ubiquitin Ligases in Breast Cancer Metastasis: A Systematic Review of Pathogenic Functions and Clinical Implications

**DOI:** 10.3389/fonc.2021.752604

**Published:** 2021-10-22

**Authors:** Yingshuang Wang, Jiawen Dai, Youqin Zeng, Jinlin Guo, Jie Lan

**Affiliations:** ^1^ Key Laboratory of Systematic Research of Distinctive Chinese Medicine Resources in Southwest China, Chengdu University of Traditional Chinese Medicine, Chengdu, China; ^2^ Chongqing Key Laboratory of Sichuan-Chongqing Co-construction for Diagnosis and Treatment of Infectious Diseases Integrated Traditional Chinese and Western Medicine, Chengdu University of Traditional Chinese Medicine, Chengdu, China; ^3^ Department of Thoracic Oncology, Department of Radiation Oncology, Cancer Center and State Key Laboratory of Biotherapy, West China Hospital, Sichuan University, Chengdu, China

**Keywords:** ubiquitination, E3 ligase, breast cancer, metastasis, systematic review

## Abstract

Female breast cancer has become the most commonly occurring cancer worldwide. Although it has a good prognosis under early diagnosis and appropriate treatment, breast cancer metastasis drastically causes mortality. The process of metastasis, which includes cell epithelial–mesenchymal transition, invasion, migration, and colonization, is a multistep cascade of molecular events directed by gene mutations and altered protein expressions. Ubiquitin modification of proteins plays a common role in most of the biological processes. E3 ubiquitin ligase, the key regulator of protein ubiquitination, determines the fate of ubiquitinated proteins. E3 ubiquitin ligases target a broad spectrum of substrates. The aberrant functions of many E3 ubiquitin ligases can affect the biological behavior of cancer cells, including breast cancer metastasis. In this review, we provide an overview of these ligases, summarize the metastatic processes in which E3s are involved, and comprehensively describe the roles of E3 ubiquitin ligases. Furthermore, we classified E3 ubiquitin ligases based on their structure and analyzed them with the survival of breast cancer patients. Finally, we consider how our knowledge can be used for E3s’ potency in the therapeutic intervention or prognostic assessment of metastatic breast cancer.

## 1 Introduction

According to the latest global cancer statistics by the International Agency for Research on Cancer, female breast cancer has overtaken lung cancer and has become the most commonly occurring cancer worldwide, which accounts for about 11.7% of all new cancer cases (1). Breast cancers are highly heterogeneous, and they are classified into subtypes as Luminal A, Luminal B, HER2 (human epithelial growth factor receptor 2) positive, and basal-like (triple negative breast cancer, TNBC), with respect to the presence or absence of hormone receptors such as estrogen receptor (ER) and progesterone receptor (PR), and one oncogenic biomarker, HER2. These molecular subtypes help determine which patients are likely to respond to targeted therapies (2). Despite having good prognosis with early diagnosis and appropriate treatment, breast cancer is still the leading cause of mortality among women (3). Breast cancer–leading deaths are mostly attributed to metastasis. As mammary epithelial cells which acquire deregulated proliferation, if these malignant cells remain contained within the ducts or lobules of breast, the patients’ survival has been reported to be nearly ~98% within 5 years. In contrast, the patients’ 5-year survival rate with distant metastases at the time of diagnosis decreased to only 23% (2). Furthermore, approximately one-third of female breast cancer patients with no lymph node involvement at the time of diagnosis will develop distal metastasis (4).

### 1.1 The Multistep Cascade of Breast Cancer Cell Alternations in Metastasis

**Figure 1:**
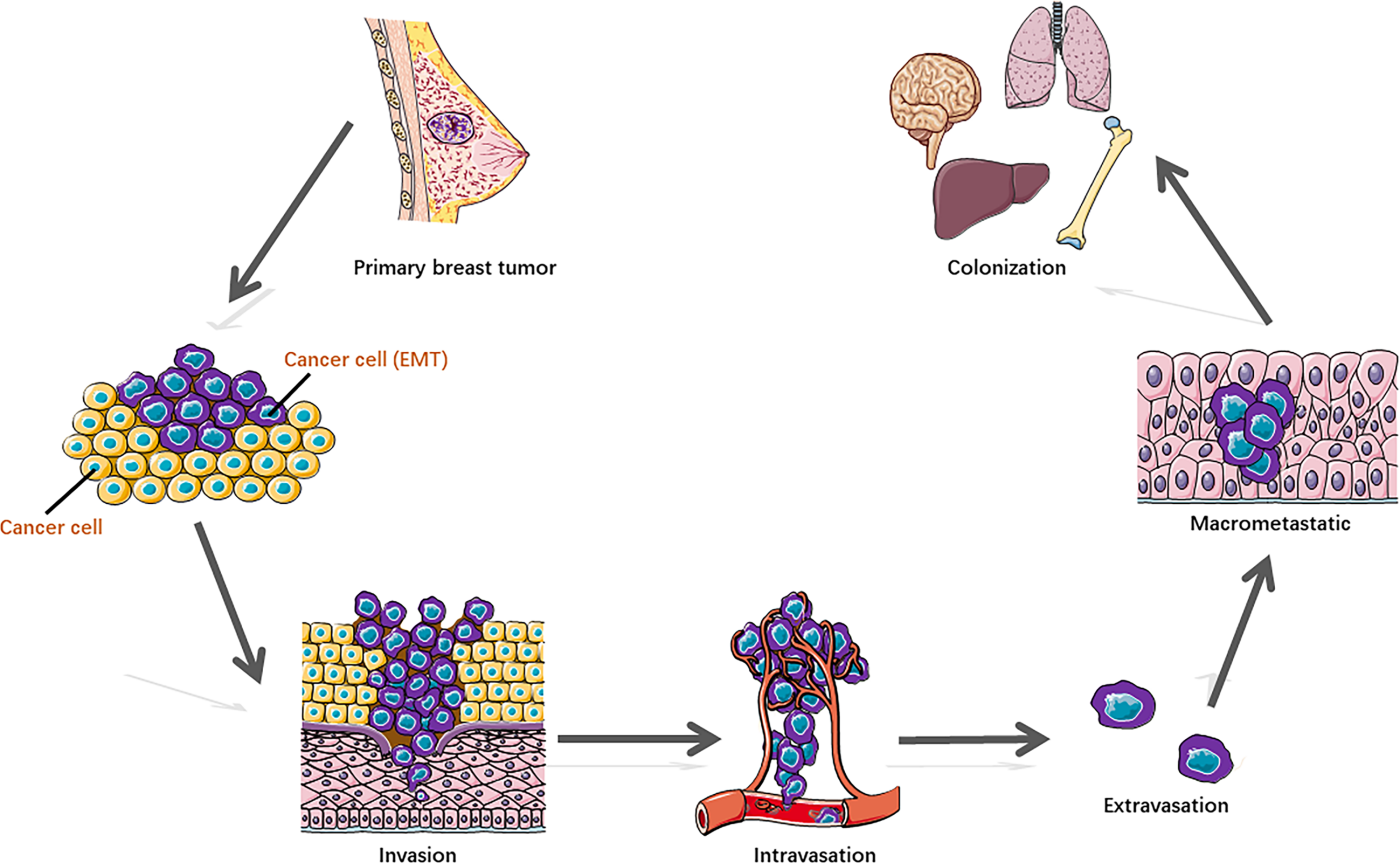
Distant metastasis colonization consists of sequential steps.

Some breast cancer cells acquire metastatic potential in a very early stage, which gains the capability to spread from the breast tissue, enter the blood or lymphatic vessels, and disseminate to distal organs, preferentially to the lung, liver, brain, and bone. Metastasis represents the multistep cascade of cancer cell alterations accompanied by structural and functional changes. It is well recognized that distant metastasis colonization consists of sequential steps (**Figure 1**) (2, 5), including cell detachment from the primary tumor site involving epithelial–mesenchymal transition (EMT) (6); migration and invasion into surrounding tissue; penetration of the basal membrane (trans-endothelial intravasation) into the vasculature of blood and/or lymphatic vessels to be circulating tumor cells (CTCs); extravasation of CTCs to secondary sites as disseminated tumor cells (DTCs) (7); dissemination to distant organs; and formation of a micro-metastatic niche and construction of macrometastases. In addition, to survive and initiate the secondary cancer foci, cells need the capability of evading immune defenses, delivering to distant sites, adapting to supportive niches such as angiogenesis (8), and inducting retro-differentiation to gain stemness (9).

### 1.2 The E3 Ubiquitin Ligases

Ubiquitination is a post-translational modification of proteins, which is essential for nearly all biological processes, including cell growth, autophagy, apoptosis, and differentiation. It is a three-step enzymatic cascade: i) A ubiquitin-activating enzyme (E1) mediates the activation of the carboxyl-terminal glycine residue of ubiquitin in an ATP-dependent manner. ii) The activated ubiquitin is then transferred to E1 followed by the transfer of ubiquitin to a thiolester of a ubiquitin-conjugating enzyme (E2) to format the thiolester linkage. iii) A ubiquitin protein ligase (E3) confers substrate specificity by recognizing the target proteins and mediating the conjugation of ubiquitin molecules to a lysine residue on the targeted protein *via* an isopeptide bond (10). E3’s ability to specifically recognize and target substrates makes it a key regulator in the ubiquitin process.

**Figure 2:**
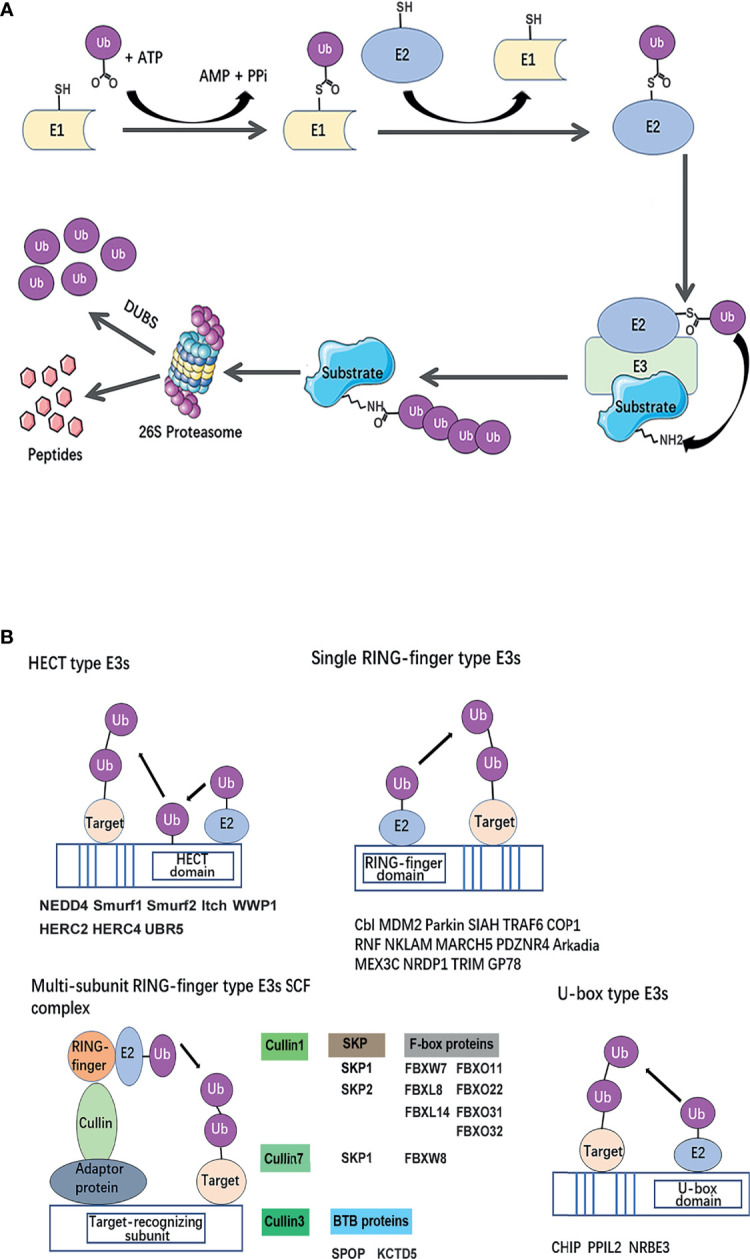
Classical mechanism and structural basis of E3 ubiquitin ligases. **(A)** The ubiquitin-proteasome system. A ubiquitin-activating enzyme (E1) mediates the activation of the carboxyl-terminal glycine residue of ubiquitin in an ATP-dependent manner. To format the thiolester linkage, the activated ubiquitin is then transferred to E1 followed by the transfer of ubiquitin to a thiolester of a ubiquitin-conjugating enzyme (E2). Ubiquitin protein ligase (E3) confers substrate specificity by recognizing the target proteins and mediating the conjugation of ubiquitin molecules to a lysine residue on the targeted protein *via* an iso-peptide bond. Polyubiquitinated substrate recognized by 26S proteasome for degradation. **(B)** Classification of breast cancer metastasis–related ubiquitin E3 ubiquitin ligases based on a structural basis. The canonical E3s are classified into two canonical types: HECT and RING. HECT E3s contains HECT domains which consist of a E2-interacting N-lobe and a catalytic Cys residue containing C-lobe involved in ubiquitin transfer. RING-type E3s mediate the direct transfer of ubiquitin from E2 to substrate, including single RING-finger- type E3s, RING-like (Ubox)-type E3s, and multi-subunit RING-finger-type E3s. The Cullin-RING ubiquitin ligase (CRL) family is composed of a multi-unit. Each type of E3s or members of E3 complexes has been summarized and listed below the schematic diagram of the relevant category.

The fate of ubiquitinated proteins is dependent on the different types of ubiquitin linkage. It has been characterized as mono-ubiquitination or poly-ubiquitinated chains. Mono-ubiquitination involves the transfer of a single ubiquitin to a substrate. E3 ligases can also connect several ubiquitin molecules together using the C-terminus of one subunit and one of the seven lysine (K) residues (K6, K11, K27, K29, K33, K48, K63) or the N-terminal methionine (M1) on the other, to form a homotypic or branched ubiquitin chain (11). The recruitment of polyubiquitinated proteins to the proteasome is a classic protein turnover pathway (**Figure 2A**). Ubiquitin-dependent proteasomal degradation involves the polyubiquitination of substrate catalyzed by E1, E2, and especially E3 (12, 13). Subsequently, polymers linked through K11 or K48 (14, 15) trigger substrate protein degradation by the ATP-dependent 26S proteasome (16).

Besides, different ubiquitin topologies adopt distinct structural conformations. Signals can be transduced by different types of ubiquitin modification, including mono-ubiquitination, multi-monoubiquitination, homotypic ubiquitin chains, and heterotypic ubiquitin chains, which send various ‘codes’ to precisely exert degradative and non-degradative functions including modification of protein trafficking, interaction, and signal transduction (17, 18). For example, linkages of homotypic K11 and K48 can drive proteasomal degradation; K27 linkages have been implicated in regulating DNA repair (19) and autoimmunity (20); K33 linkages were proposed to regulate trafficking; and mono-ubiquitination can prevent protein interactions (21). SMAD4 (SMAD family member 4) engages its signal partner SMAD2 (SMAD family member 2) after removing its own mono-ubiquitination (18). In addition, the mono-ubiquitination of histone H2A promotes transcriptional silencing (22), while the mono-ubiquitination of histone H2B mediates transcriptional elongation (23).

The canonical E3s are classified into two types: Homologous to the E6AP Carboxyl Terminus (HECT) family (24) and Really Interesting New Gene (RING) finger family (25). HECT E3 ligases were named accordingly to identify protein E6AP (E6-associated protein), and they are characterized by a conserved C-terminal ~350 aa HECT domain and various N-terminal substrate-binding domains (26). HECT E3 ligases mediate a two-step ubiquitin transfer process, in which ubiquitin is firstly transferred from the E2-Ub intermediate to the E3 active cysteine residue before transferring to the substrate lysine residue (**Figure 2B**) (27). In contrast, The RING core is a small domain of 40–70 residues. A cross-braced pattern of conserved cysteine and histidine residues coordinating two zinc ions maintains the native fold. The RING domain brings the E2∼Ub conjugate into the proximity of the substrate bound *via* substrate-recognition domains (**Figure 2B**). RING E3s are further divided into two subtypes: single RING and multi-unit RING family. The Cullin-RING ubiquitin ligase (CRL) family is composed of a multi-unit. CRLs utilize Cullin proteins as a central scaffold which binds to a RING-box protein (Rbx) and an adaptor protein–substrate receptor complex through its C- and N-termini. Most recognized CRLs are known as the SCF (Skp-Cullin-F-box) complex, in which Cullin interacts with Skp (S-phase kinase associated protein) proteins and utilizes various F-box proteins, to recruit substrates and initiate ubiquitin ligation (28). Besides, U-box E3s are also categorized as RING-type E3s, but their molecular structure subtly differs in that zinc-bound sites are replaced by a hydrophobic core (29) (**Figure 2B**).

E3s target a broad spectrum of substrates. In cancer, the aberrant functions of E3s are linked to deregulated oncoproteins or tumor suppressors and affect the biological behavior of cells. Many E3s have been reported to be associated with breast cancer metastasis over the past few decades. In this review, we provided an overview of E3 ubiquitin ligases that have been found to be deregulated in breast cancer metastasis and summarized the multistep cascade of breast cancer cell alterations in metastasis, in which these E3 ubiquitin ligases are involved. Furthermore, we classified E3s based on their structures and analyzed the correlation of E3s with the survival of breast cancer patients. Finally, we considered their potency to the therapeutic intervention or prognostic assessment of metastatic breast cancer.

## 2 Methods

### 2.1 Search Strategy and Selection Criteria

The literatures involved in this study were searched from the databases PubMed and Medline (last search updated on January 1st, 2021). The key words used in the searching were “E3 ubiquitin”, “breast cancer” and “Metastasis” or “dissemination”/“EMT”/“invasion”/“migration”/“intravasation”/“CTC”. All the searching results were imported in the Endnote software to eliminate duplicates.

By scanning the titles and abstracts and further reading the full text, the irrelevant and retracted papers were excluded. The reference list of all selected articles was scanned to identify potentially relevant reports. The search results followed these including and excluding criteria:

*Including criteria*: 1) research limits to the E3 ubiquitin ligases; 2) research related to human breast cancer; 3) research focused on metastasis or steps in the metastasis mechanisms of breast cancer; 4) studies provided sufficient experimental evidence or clinical data to support thesis; 5) peer-reviewed and formally published original literatures

*Excluding criteria*: 1) research of the regulation of E3 ubiquitin ligases; 2) research of ubiquitin-like modifiers including SUMOylation; 3) retracted articles; 4) reviews or letter to editors.

### 2.2 Data Extraction

The above searches were performed and reviewed by two authors independently. The following items were recorded from each study: E3 name, gene ID, substrate, role in breast cancer metastasis, cellular function, signal pathway, verification by cell biological experiments, breast cancer cell lines, and clinical significance (expression difference and survival analysis). These records were cross-checked and double- checked by another author.

### 2.3 Survival Analysis

The association between the specific E3s’ ubiquitin ligase expression and survival in breast cancer was first analyzed using the PrognoScan database (30) as in previously mentioned methods (31). Then, we used a Kaplan–Meier plotter (32) to validate and illustrate as a Kaplan–Meier plot, in which the distant metastasis–free survival (DMFS) curves for high (red) and low (black) expression groups dichotomized at the optimal cut-point were plotted. The logrank P-value and the hazard ratio with 95% confidence intervals were calculated. The threshold was adjusted to logrank P‐values at <0.05.

## 3 Results

### 3.1 Description of Collected Studies

**Figure 3:**
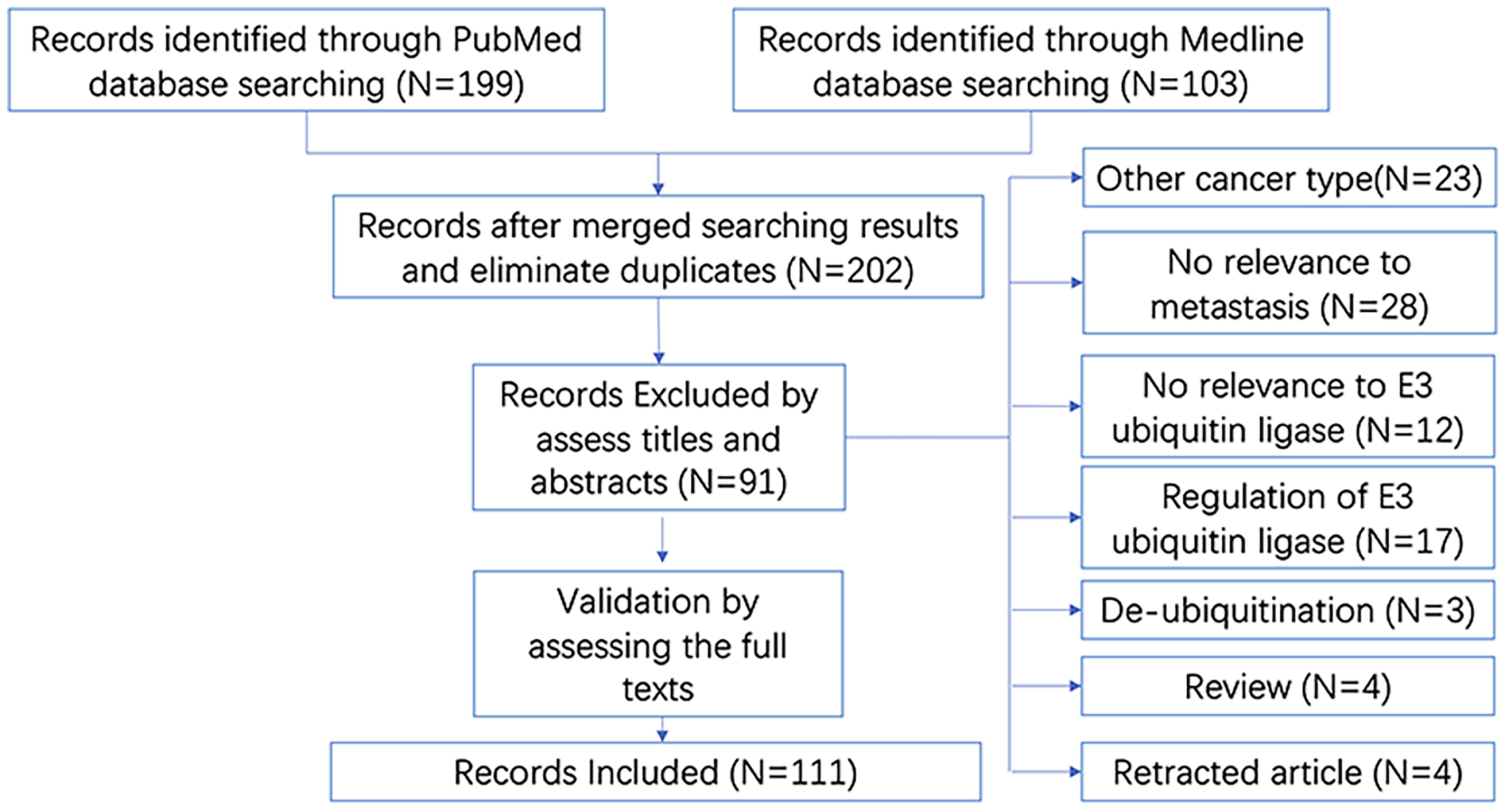
Brief flow chart for literatures searching and selection. “N” refers to “number of studies”.

As shown in the brief flow chart (**Figure 3**), the records on E3 ubiquitin ligases involved in breast cancer metastasis were screened from both the PubMed (N = 199) and Medline (N = 103) databases. After duplicate elimination, 202 records remained. By reading the titles and abstracts of these records, we excluded 91 literatures for the following reasons: studies in other cancer types (N = 23); irrelevant to metastasis (N = 28) or irrelevant to E3 ubiquitin ligase (N = 12); focused on the regulation of E3 ubiquitin ligase (N = 17) or de-ubiquitination (N = 3); and reviews (N = 4) and retracted articles (N = 4). Other studies had been validated by reading the full texts. Finally, a total of 111 literatures were included in our analysis.

### 3.2 Roles of E3 Ubiquitin Ligases Played in the Multiple Steps of Breast Cancer Metastasis

We further extracted key information from these literatures, including E3 name, gene ID, substrate, roles in breast cancer metastasis, cellular function (experimentally verified), signal pathway, breast cancer cell lines, and clinical significance. This detailed information of 54 E3 ubiquitin ligases/ligase complexes in total have been summarized in **Table 1**. By their molecular roles’ participation in the multiple steps of breast cancer metastasis, we briefly presented them as follows.

**Table 1:** E3 ubiquitin ligases that played important roles in the multiple steps of breast cancer metastasis .

E3	Substrate	Inhibit/Promote Metastasis	Cellular Function	Molecular Pathway	References
Arkadia	Ski	Inhibit	EMT	TGF-beta	(33)
ASB13	SNAI2	Inhibit	Migration	Hippo–YAP	(34)
BCA2	Autoubiquitination	Promote	Migration and invasion	EGFR	(35, 36)
Cbl	FAK and EGFR	Promote/inhibit	Cell detachment/EMT and migration	FAK, RANKL/RANK EGFR-ERK/Akt	(37–40)
CHIP	Pfn1	Promote	Migration	ROCK1/Pfn1	(41, 42)
COP1	c-Jun	Inhibit	Migration	ETV1, GSK3β/c-Jun	(43, 44)
Cullin1	N/A	Promote	EMT, migration, invasion, and tube formation	PI3K/AKT, NF-κB	(45)
Cullin3/SPOP	PR, Erα	Inhibit	Migration	PR, ERα	(46, 47)
Cullin3/KCTD5	N/A	Promote	Migration and invasion	TRPM4	(48)
Cullin7	N/A	Promote	Invasion	N/A	(49)
FBXW7	NICD1/Notch1	Promote/inhibit (indirect evidence)	Migration and invasion	NOTCH/p62	(50, 51)
FBXL8	N/A	Promote	Migration and invasion	CCND2/IRF5	(52)
FBXL14	CDCP1	Inhibit	EMT, migration, and invasion	PI3K/AKT	(53)
FBXO11	SNAI1	Inhibit	Emt	SNAI1 and p53/p21/BCL2	(54, 55)
FBXO22	HDM2	Inhibit	Migration and invasion	p53/p21, SNAIL	(56)
FBXO31	Slug	Inhibit	Invasion	N/A	(57)
FBXO32	KLF4	Promote	EMT, migration, and invasion	N/A	(58)
GP78	HSPA5	Inhibit	Migration and invasion	N/A	(59, 60)
HACE1	Rac1	Inhibit	Migration and invasion	N/A	(61)
HectD1	ACF7	Inhibit	EMT, migration, and invasion	N/A	(62)
HERC2	BRCA1	Promote (indirect evidence)	Invasion (indirect evidence)	BRCA1	(63)
HERC4	LATS1	Promote	Migration and invasion	LATS1	(64)
HRD1	IGF-1R	Inhibit	EMT, migration, and invasion	IL6/NF-κB	(65)
ITCH	c-Jun, p73, p63, and ErbB4; RASSF1A; histone H1.2	Promote	EMT and invasion	Hippo	(66–68)
MARCH5	N/A	Promote	Migration and invasion	Mitochondrial	(69)
MDM2	P53; RB; Foxo3a	Promote	EMT, migration, and invasion	p53/p21	(70–73)
MEX3C	PTEN	Promote	EMT	TWIST1, SNAI1, and YAP1	(74)
NEDD4	Robo1	Promote	Migration and invasion (indirect evidence)	FAK and Src	(75–77)
NKLAM	N/A	Inhibit	Tumor immunity	NK killing activity	(78)
NRBE3	RB	Promote	Migration and invasion	E-cadherin	(79)
NRDP1	ErbB3 and ErbB4	Inhibit (indirect evidence)	Migration and invasion (indirect evidence)	ER	(80)
Parkin	HIF-1α	Inhibit	Migration and invasion	HRE/VHL	(81)
PDZRN4	Kidins220 (predict)	Inhibit	Migration and invasion	N/A	(82)
PPIL2	SNAI1	Inhibit	EMT, migration, and invasion	SNAI1	(83)
RNF8	TWIST	Promote	EMT, migration, and invasion	TWIST; GSK3β	(84, 85)
RNF20	Histone H2B	Promote (luminal)/inhibit (in basal-like)	Migration	ER (in luminal) and NF-kB (in basal-like)	(86, 87)
RNF144A	HSPA2	Inhibit	Migration and invasion	HSPA2	(88)
RNF208	Vimentin	Inhibit	Migration and invasion	Vimentin	(89)
SIAH1/2	p27	Promote	Migration and invasion	Rb	(90)
SKP2	AKT	Promote	Indirect evidence	PI3K/AKT	(91)
SCF-JFK	ING4	Promote	EMT, invasion, and angiogenesis	NF-κB	(92)
β-Trcp	PRLr/βCatenin	Promote	EMT, migration, and invasion	PRL,Wnt	(93, 94)
Smurf1	RhoA p120-catenin and TRAF4	Promote	EMT, migration, and invasion	TGFβ	(95–99)
Smurf2	Smurf1, CNKSR2	Promote/inhibit	Migration and invasion	TGFβ, PI3K/AKT	(100–104)
TRAF6	H2AX	Promote	Migration and invasion	HIF1α	(105, 106)
TRIM8	Erα	Inhibit	Migration and invasion	Erα	(107)
TRIM11	Erα	Promote	Migration	Erα	(108)
TRIM44	N/A	Promote	Migration	NF-κB	(109)
TRIM47	N/A	Promote	EMT, migration, and invasion	PI3K/Akt	(110)
UBR5	N/A	Promote	EMT, migration, and invasion	STAT3, TGFα, P38MAPK	(111, 112)
UBR7	H2B	Inhibit	EMT, migration, and invasion	Wnt/β-catenin	(113)
WWP1	CXCR4 (indirect evidence)	Inhibit	Migration	TGFβ/Smad	(114)
xIAP	TAK1	Promote	Invasion	TGFβ/Smad	(115)
UBE2O	AMPkα2	Promote	EMT, migration, invasion, and stemness	AMPK/mTOR	(116)

ASB13, ankyrin repeat and SOCS box containing 13; BCA2, breast cancer associated gene 2; Cbl‐b, Cbl proto-oncogene B; CHIP, STIP1 homology and U-box containing protein 1; CXCR4, C-X-C motif chemokine receptor 4; SPOP, speckle type BTB/POZ protein; KCTD5, K^+^ channel tetramerization domain 5; FBXW7, F-box and WD repeat domain containing 7; FBXL8, F-box and leucine rich repeat protein 8; FBXL14, F-box and leucine rich repeat protein 14; FBXO11, F-box protein 11; FBXO22, F-box protein 22; FBXO31, F-box protein 31; FBXO32, F-box protein 32; GP78, autocrine motility factor receptor; HACE1, HECT domain and ankyrin repeat containing E3 ubiquitin protein ligase 1; HECTD1, HECT domain E3 ubiquitin protein ligase 1; HERC2, HECT and RLD domain containing E3 ubiquitin protein ligase 2; HERC4, HECT and RLD domain containing E3 ubiquitin protein ligase 4; MARCHF5, membrane associated ring-CH-type finger 5; MEX3C, mex-3 RNA binding family member C; NKLAM, Natural killer lytic-associated molecule; NRBE3, New RB E3 ubiquitin ligase; NRDP1, also known as RNF41, ring finger protein 41; Parkin, parkin RBR E3 ubiquitin protein ligase; PDZRN4, PDZ domain containing ring finger 4; PPIL2, peptidylprolyl isomerase like 2; RNF, RING Finger Protein; RASSF1, Ras association domain family member 1; SIAH1/2, siah E3 ubiquitin protein ligase 1 and 2; SKP2, S-phase kinase associated protein 2; SCF, Skp-Cullin-F-box) complex; β-Trcp, beta-transducin repeat containing E3 ubiquitin protein ligase; SMURF1 and SMURF2, SMAD specific E3 ubiquitin protein ligase 1 and 2; TRAF6, TNF receptor associated factor 6; TRIM8, TRIM11, TRIM44, TRIM47: tripartite motif containing 8, 11, 44 and 47; UBR5 and UBR7, ubiquitin protein ligase E3 component n-recognin 5 and 7; TWIST, twist family bHLH transcription factor 1; WWP1, WW domain containing E3 ubiquitin protein ligase 1; xIAP, X-linked inhibitor of apoptosis; UBE2O, ubiquitin conjugating enzyme E2 O; N/A, Not Available in the reference literature.

#### 3.2.1 Breast Cancer Cell Epithelial–Mesenchymal Transition

EMT is thought to be a hallmark in tumor metastasis (117, 118). By the loss of cell–cell conjunction, the epithelial cells acquire migratory, invasive properties and trans-differentiate to mesenchymal phenotypic cells (9). Carcinoma cells undergoing EMT can escape from primary tumor sites, enter the circulation, and then move out to invade distant sites where secondary tumors or metastases begin to form (117, 119).

Not all breast cancer cells are typical EMT cells. In a study of breast cancer aggressiveness, by profiling EMT-like subclones from MCF-7 breast cancer cells based on EMT properties, comparing their gene-expressing differences with other non-EMT-like subclones, FBXO11 (F-box protein 11, a member of the E3 ubiquitin ligase complex) was screened out. It is concluded that FBXO11 is a candidate molecular alternative to the canonical EMT-dependent aggressiveness in highly differentiated luminal tumors (54). The specific roles suggest that E3 ligase FBXO11 targets transcription factor SNAI1 (snail family transcriptional repressor 1) protein which induces EMT, for ubiquitination-dependent degradation (55), and regulates the p53/p21/BCL2 pathway (54).

In cancer cells, the loss of E-cadherin results in EMT and contributes to increased metastasis and chemoresistance (120, 121). The absence of the ubiquitin protein ligase E3 component n-recognin 5 (UBR5) has been characterized to trigger aberrant EMT in TNBC, principally *via* an abrogated expression of E-cadherin, which resulted in severe lung metastasis in UBR5 knockout mice. The E-cadherin transcription repressors slug, twist, and zinc finger E-box-binding homeobox 1/2 (ZEB1/2) are overexpressed in multiple drug-resistant (MDR) breast cancer cells, making them more metastatic (122). The E3 ubiquitin ligase Casitas B lymphoma-b (Cbl-b) was reported to prevent tumor metastasis by maintaining the epithelial phenotype in MDR breast cancer cells. Cbl-b inhibits MDR breast cancer cell migration by specifically targeting epidermal growth factor receptor (EGFR) ubiquitination-dependent degradation, which prevents metastatic breast cancer cell EMT by inhibition of the EGFR-ERK/Akt-miR-200c-ZEB1 axis (37).

Not only restricted in the degradation of substrates, an RNA-binding E3 ligase, mex-3 RNA binding family member C (MEX3C), catalyzed protein PTEN (phosphatase and tensin homolog) K27-linked polyubiquitination, leading to its enzymatic functions’ switch to serine/threonine phosphatase activity (74). Consequently, switched PTEN promotes EMT.

The roles of E3 ubiquitin ligases played in breast cancer cell EMT include regulating transcription factors in EMT-inducing gene expression, abrogated expressing of key proteins, and affecting the activities of substrate proteins. The specific modification is not only as an E3 ubiquitin ligase to influence protein stability but also to induce the change of protein catalytic activities.

#### 3.2.2 Breast Cancer Cell Invasion and Migration

Escaping from the surrounding tissues of the primary tumor, invading the blood or lymphatic vessels (intravasation), and migrating are essential steps in breast cancer metastasis. In this analysis, most of the E3 ubiquitin ligases (50 out of 54) have been detected to promote or inhibit the invasion and migration of breast cancer cells. Ubiquitin E3 ligases for tumor suppressors play important roles in tumorigenesis. The E3 ligase MDM2 has been characterized to target both tumor suppressor p53 and RB for proteasomal degradation, which promotes breast cancer cell invasion and migration *via* the p53/p21 pathway (70, 71). Nedd4 and carboxyl terminus of Hsc-70-interacting protein (CHIP) are two E3 ubiquitin ligases which function as regulators of tumor suppressor PTEN (123, 124). CHIP has been confirmed to promote breast cancer cell migration (41); the role of Nedd4 in breast cancer cell migration is not clear.

In the migration process, β-catenin and E-cadherin are two key proteins that function in cell–cell adhesion. SCF^βTrcp^ has been characterized to be the E3 ligase complex responsible for β-catenin degradation (93). As for E-cadherin, MDM2 has also been identified as responsible for its ubiquitin-dependent degradation (71). The E3 ligase new RB-E3 ligase protein (NRBE3) promotes breast cancer cell migration *via* E-cadherin but is not dependent on its E3 ligase property (79). Although it has been speculated that UBR5 might regulate E-cadherin through targeting substrates for proteasome-dependent degradation, both *in vivo* and *in vitro* ubiquitin assays were required for validation (111).

#### 3.2.3 Breast Cancer Cell Stemness

Breast cancers are heterogenous. Although breast cancer stem cells (BCSCs) account for a very small percentage, they have the greatest ability of self-renewal and potential of unlimited differentiation capacity into heterogeneous tumor cell populations, all of which contribute to regenerate tumor at original or distant sites *in vivo.* Cancer cell stemness properties can be identified by several stem cell markers such as CD34, CD44, CD123, CD133, Oct4, Sox2, Nanog, ABCG2, and MYC and the conduction of cell stemness sphere assays (116, 125).

Not only is MYC a stem cell marker; it is also a well-characterized oncoprotein that is upregulated in 30–50% of breast cancer patients. MYC is regulated by AMPKα (AMP-activated protein kinase alpha subunit)/mTORC1 (mechanistic target of rapamycin kinase) axis. Ubiquitin-conjugating enzyme E2O (UBE2O), a large E2 ubiquitin-conjugating enzyme that represents both E2 and E3 ligase activities, is found to promote AMPKα ubiquitination and degradation and then to activate the mTORC1-MYC signal pathway in breast cancer cells. By detection of stem cell markers and observation of cell stemness sphere formation, it is suggested that UBE2O endowed breast cells with cancer stemness properties (116).

In a study of the characteristics of BCSCs by selectively sorting cells with stem cell markers, another E3 ligase, Cbl, has been found to be involved in maintaining cancer cell stemness. EGFR is important for cancer stem cell maintenance and metastasis. Its turnover relies on the ubiquitin pathway. Cbl-c, a member of Cbl family, can target EGFR for k-63 linked ubiquitination and lysosomal degradation. By interfering with the binding of EGFR and its E3 ubiquitin ligase Cbl-c, a membrane protein sarcoglycan epsilon (SGCE) inhibits Cbl-c ubiquitin ability and stabilizes EGFR and then promotes breast cancer cell stemness (38).

Whether it is in regulating stem property–maintaining key proteins, or in the analysis of BCSCs’ characteristics, E3 ubiquitin ligases have been found to play roles in regulating the pluripotency of BCSCs.

#### 3.2.4 Angiogenesis

In the process of metastasis, to survive and initiate the secondary cancer foci, cells need the capability of adapting to supportive niches such as angiogenesis (8). Two members of E3 ligases complex were found to be functional in angiogenesis.

The Skp–CUL1–F-box ubiquitin ligase complex is one of the best-characterized multi-subunit RING finger complexes composed of four subunits. F-box protein 42 (Fbxo42, also known as JFK) is one of the F-box family proteins. It is demonstrated that JFK targets ING4 for ubiquitination and degradation through the assembly of an SCF^JFK^ ubiquitin ligase. ING4 is a member of the inhibitor of growth (ING) protein family, defined as tumor suppressors by directly interacting with p53 and promotes the transactivation of p53 and negatively regulates NF-κB-responsive gene transcription. The NF-κB pathway regulates the expression of several prominent pro-angiogenic factors, including IL-6, IL-8, CCL5, and COX-2. Degradation of ING4 by E3 ligase SCF^JFk^ results in the destabilization of NF-κB signaling and promotes angiogenesis. In breast cancer animal models, it was also observed that JFK-mediated metastasis takes the roots of lungs (92).

Another member of the SCF complex is Cullin1. In the tube formation assay, the knockdown of Cullin1 significantly decreased the number of complete tubule structures formed by human umbilical vein endothelial cells (HUVECs) *in vitro*. In the tail vein metastasis animal model, MDA-MB-231 cells with Cullin1 stable knockdown showed reduced vascularization and micro-vessels in matrigel plug (45). Cullin1 regulates the zeste 2 polycomb repressive complex 2 subunit (EZH2), which enhances cytokine expression through the NF-κB pathway. The cytokine expression further results in aggravating the breast cancer cell metastasis through the PI3K–AKT–mTOR signaling pathway. Due to the lack of ubiquitin assays, it is unclear whether EZH2 is the substrate protein of Cullin1. Besides, in the SCF E3 ubiquitin complexes, Cullin1 often acts as a ‘scaffold’ protein; what the target-recognizing subunit in this complex is also needs to be clarified further.

Both JFK and Cullin1 regulated angiogenesis in breast cancer metastasis, through the NF-κB pathway. It is noticed that three other E3 ubiquitin ligases, hydroxymethylglutaryl-coenzyme A reductase degradation protein 1 (HRD1) (65), ring finger protein 20 (RNF20) (86), and tripartite motif containing 44 (TRIM44) (109), were also found regulating the NF-κB pathway. We need to pay attention to whether they are responsible for angiogenesis in breast cancer metastasis in addition to their existing roles.

#### 3.2.5 Immunity Response/Rescue

The multiple steps of metastasis rely on reciprocal interactions between breast cancer cells and the microenvironment. Immune cells and their mediators are known to facilitate metastasis within the microenvironment (126). Natural killer (NK) cells play an essential role in the defense against viruses or microbial pathogens and malignancies produced by the body itself. In the process of immune response, NK cells execute anti-pathogen or anti-tumor activities that rely on the direct cytolytic activity of these cells and produce various cytokines (127). An E3 ubiquitin ligase, the natural killer lytic-associated molecule (NKLAM), has been characterized to play a major role in the cytolytic activity of NK cells and to control tumor development, dissemination, and distant metastasis *in vivo*. The target substrate of NKLAM in NK cells has not been directly determined yet (78).

Recently, ubiquitin protein ligase E3 component N-recognin 5 (UBR5) has been found to induce a CD8+ T cell–mediated immune response. The loss of UBR5 in breast cancer cells causes the appearance of certain putative immunogens’ strong CD8+ T cell–mediated response in a paracrine manner (112). UBR5 has been previously found to be involved in TNBC metastasis (111). According to these two studies, the loss of UBR5 caused reduced angiogenesis and triggered aberrant EMT depending on the EMT regulators’ inhibitor of DNA binding 1 and 3 (ID1 and ID3), which limited the metastasis of breast cancer. Although it was indicated that UBR5 executed its biological function principally *via* abrogated expression of E-cadherin, its specific ubiquitin substrate still needs validation through both *in vivo* and *in vitro* ubiquitin assays.

More and more evidences suggest that E3 ubiquitin ligases participate in the regulation of immunosuppression. Whether E3 can be used as combined targets of tumor immunotherapy in the future needs further research (126, 128).

Thus, the identifying regulators of each step in the process above should provide insights into the mechanisms that control breast cancer metastasis and hence patient survival.

### 3.3 Classification of Breast Cancer Metastasis–Related E3s Based on the Structure

Ubiquitination is a ubiquitous form of post-translational modification of proteins. In this process, E3s specifically bind to the substrate proteins, mediate the ligation of ubiquitin molecules and affect the specific proteins' turnover and functions. Thus, it is important to clarify the mechanism in target drug research and development (129). Do the mechanisms of these identified breast cancer metastasis E3 ligases have commonalities?

We classified the previously summarized E3 ligases of classical families based on their structures. The results suggested that the E3s involved in breast cancer metastasis belong to diversified classes, such as the HECT family, RING family, and U-box family (**Figure 2B**). There are eight E3 ligases that belong to the HECT family and are further classified into three subfamilies, including SMAD-specific E3 ubiquitin protein ligase 1 (Smurf1), SMAD-specific E3 ubiquitin protein ligase 2 (Smurf2), itchy E3 ubiquitin protein ligase (Itch) and WW domain–containing E3 ubiquitin protein ligase 1 (WWP1), NEDD4, belonging to the NEDD4 family, which contains a WW domain, C2 domain, and HECT domain; HECT and RLD domain which contains E3 ubiquitin protein ligases 2 and 4 (HERC2 and HERC4), as the HERC family, which contains the common RCC1-like domain (RLD) and HECT domain; and UBR5, as the “other” family, which contains a UBA domain, zinc finger domain, and HECT domain.

RING E3 ligases are further divided into two subtypes: single RING and multi-unit RING family. Several E3 ligases belong to single RING type (listed in **Figure 2B**, single RING family), performing a single-step ubiquitin transfer from the E2-Ub to the substrate, which work as allosteric activators. As for multi-subunit RING type, The Cullin-RING ubiquitin ligase (CRL) family is composed of a multi-unit. Most recognized CRLs are known as the SCF (SKP-Cullin-F-box) complex, in which Cullin1 interacts with Skp1 or Skp2 and utilizes various F-box proteins (shown in **Figure 2B**, F-box proteins) to recruit substrates and initiate ubiquitin ligation. Cullin7 also forms a SCF complex with SKP1 and F-box and WD repeat domain containing 8 **(**FBXW8). Cullin3-RBX1 (Ring-box1) E3 ubiquitin ligase complex requires BTB (Bric-a-brac-Tramtrack-Broad complex) domain protein as an adaptor. Two BTB proteins, speckle-type BTB/POZ protein (SPOP) and K^+^ channel tetramerization domain 5 (KCTD5), forms a complex with Cullin3, respectively.

Three E3 ubiquitin ligases, CHIP, NRBE3, and peptidylprolyl isomerase like 2 (PPIL2) belong to the U-box family, which contains a U-box domain. U-box E3s are also categorized as RING-type E3s, but their molecular structure subtly differs in that zinc-bound sites are replaced by a hydrophobic core (29).

There are still many E3 ligases that cannot be characterized into classical types. Ankyrin repeat and SOCS box containing 13 (ASB13) belongs to the ankyrin repeat and suppressor of cytokine signaling (SOCS) box (Asb), which contains six-ankyrin repeat domain (34, 130). The UBR-box is a 70-residue zinc finger domain present in the UBR family of E3 ubiquitin ligases. Unlike UBR5, which also contains an HECT domain, the structures responsible for UBR7 executing its E3 role need to be verified by *in vitro* ubiquitin assays (113, 131, 132). Intriguingly, UBE2O, an E2/E3 hybrid ubiquitin-protein ligase, displays both E2 ubiquitin conjugating enzyme and E3 ubiquitin ligase activities (116, 133). Compared to classical types, the specific catalytic mechanism of several non-classical E3s will need more efforts to be figured out in the future.

### 3.4 Analysis and Summary of E3 Ubiquitin Ligases Significantly Correlated With Breast Cancer Patients’ Survival

Since the E3s are involved in breast cancer metastasis, are they associated with patients’ survival? Through the analysis of literatures, we found that out of the 54 E3 ligases, 31 have been reported to be related to the survival of patients (**Table 2**, from “ASB13” to “UBE2O”). The other 23 E3 ubiquitin ligases lack clinical data to determine whether they have clinical significance. Although some of them have verified the specific roles involved in metastasis by cellular experiments and animal models, these E3 ligases’ relationships with breast cancer patients’ survival need to be elucidated. Therefore, we used publicly available clinical data to conduct survival analysis (only in which the literature were claimed to have been verified by molecular and animal experiments).

**Table 2:** E3s are significantly correlated with patients’ survival.

E3s	Clinical Significance								
	Population	DetectionType	Method of Detection	Expressing Difference	Categorization	Survival Analysis	HR	95%CI	Logrank P
ASB13	NA	mRNA	RNA-seq	Expressing difference	High *vs*. Low	OS	0.67	NA	5.7e–09
BCA2	3,554	mRNA	RNA-seq	Expressing difference (in subtype cell lines)	High *vs*. Low	OS (in subtypes)	LuminalA: 1.21	1–1.46	0.046
							LuminalB: 1.41	1.14–1.75	0.0018
							Basal: 1.25	0.96–1.64	0.1
							HER2+: 1.54	1.01–2.34	0.044
Cbl‐b	292	Protein	IHC	Expressing difference	Positive *vs*. Negative	OS, DFS	OS: 0.550	0.341–0.888	0.013
							DFS: 0.616	0.414–0.917	0.016
COP1	105	Protein	IHC	Expressing difference	Positive *vs*. Negative	OS, RFS	RR: 0.65	0.149–6.732	P < 0.001
Cullin7	103	Protein	IHC	Expressing difference	Positive *vs*. Negative	OS	NA	NA	P < 0.05
FBXL14	1,764	mRNA	RNA-seq	Expressing difference	High *vs*. Low	RFS	NA	NA	P < 0.0001
FBXO11	OS: 1,402 RFS: 3,951 MFS: NA	mRNA	RNA-seq	Expressing difference	High *vs*. Low	OS,	OS: 1.37	1.08–1.74	0.01
						RFS	RFS: 1.46	1.31–1.63	P < 0.0001
						MFS	MFS: 0.64	NA	0.04
FBXO22	410	Protein	IHC	Expressing difference	Positive *vs*. Negative	OS, DFS	OS: 0.604	0.398–0.918	0.018
							DFS: 0.536	0.315–0.912	0.021
GP78	108	Protein	IHC	Expressing difference	Positive *vs*. Negative	OS, DFS	NA	NA	<0.001
HACE1	1,764	mRNA	RNA-seq	Expressing difference	High *vs*. Low	RFS	1.40	1.20–1.64	<0.0001
HectD1	1,864	mRNA	RNA-seq	Expressing difference	High *vs*. Low	OS	NA	NA	1.00e–16
HERC4	161	mRNA	RNA-seq	Expressing difference	High *vs*. Low	OS	NA	NA	0.029
HRD1	170	Protein	IHC	Expressing difference	Positive *vs*. Negative	OS	NA	NA	<0.01
ITCH	OS: 1,115	mRNA	RNA-seq	Expressing difference	Low *vs*. High	OS, RFS, DMFS, PPS	OS: 1.47	1.15–1.87	0.0016
	RFS: 3,455;						RFS: 1.39	1.24–1.56	2.2e–08
	DMFS: 1,609						DMFS: 1.39	1.14–1.71	0.0013
	PPS: 351						PPS: 1.3	1–1.68	0.047
MARCH5	RNA: 1,081; IHC: 65	mRNA Protein	RNA-seq IHC	Expressing difference	High *vs*. Low Positive *vs*. Negative	OS	NA	NA	0.048 0.029
Parkin	RNA: 3,951 IHC: 168	mRNA Protein	RNA-seq IHC	Expressing difference	High *vs*. Low Tumor *vs*. non-tumor	RFS, DMFS	NA	NA	2.4e–13 0.0090
PDZRN4	81	mRNA Protein	RNA-seq IHC, WB	Expressing difference	Low *vs*. High	OS, DFS	OS: 1.663	1.013–2.731	0.044
							DFS: 1.840	1.126–3.007	0.015
RNF8	IHC: 202 RNA: 3,315	Protein mRNA	IHC RNA-seq	Expressing difference	Low *vs*. High	OS, RFS, DMFS, PPS	OS: 1.31	1.02–1.68	0.035
							RFS: 1.15	1.03–1.29	0.013
							DMFS: 1.43	1.17–1.76	0.00056
							PPS: 1.22	0.93–1.6	0.16
RNF144A	166	Protein	IHC	Expressing difference	Low *vs*. High	OS, DMFS	NA	NA	<0.05
RNF208	3,951	mRNA	qRT-PCR RNA-seq	Expressing difference in subtype	Low *vs*. High	RFS	0.76	0.69–0.85	<0.001
SIAH2	235	Protein	IHC	Expressing difference	Low *vs*. High	OS (ER+)	0.68	0.52–0.89	<0.005
SKP2	80	Protein	IHC	Expressing difference in subtype	Low *vs*. High	OS (Her2+)	NA	NA	0.0002
SCF-JFK	NA	mRNA	RNA-seq	Expressing difference	Low *vs*. High	OS	LuminalA: 0.94	NA	0.035
							Basal: 7.24		0.035
TRAF6	212	Protein	IHC	Expressing difference	Low *vs*. High	DMFS	NA	NA	<0.001
TRIM8	IHC: 91 RNA: NA	Protein mRNA	IHC RNA-seq	Expressing difference	Low *vs*. High	OS	All type: 0.69	0.58–0.81	3.8e–06
							ER+: 0.71	0.53–0.96	0.025
TRIM11	NA	mRNA	RNA-seq	Expressing difference	Low *vs*. High	OS	OS: 1.63	1.2–2.22	0.005
						RFS	RFS: 1.57	1.01–2.42	0.027
TRIM44	129	Protein	IHC	Expressing difference	Low *vs*. High	OS	NA	NA	0.031
						DMFS	NA	NA	0.027
UBR5	IHC: 54 RNA: NA	Protein mRNA	IHC RNA-seq	Expressing difference	Tumor *vs*. non-tumor Low *vs*. High	mRNA OS	NA	NA	0.011
UBR7	47	mRNA	RNA-seq	Expressing difference	Low *vs*. High	DMFS	0.31	0.1–0.98	0.036
WWP1	33 and 179	Protein	IHC	Expressing difference	Positive *vs*. Negative	DMFS	NA	NA	<0.05
UBE2O	RNA: 3,951 IHC: 50	mRNA Protein	RNA-seq IHC, WB	Expressing difference	Low *vs*. High	OS, DMFS	IHC OS: NA	NA	P < 0.05
							mRNA OS: 1.63	1.3–2.04	1.5e–05
							DMFS: 1.54	1.25–1.89	4e–05
Arkadia	2,765	mRNA	RNA-seq	NA	Low *vs*. High	DMFS	0.73	0.62–0.87	0.00046
MDM2	2,765	mRNA	RNA-seq	Expressing difference	Low *vs*. High	DMFS	All type: 0.81	0.69–0.96	0.015
							LuminalA: 1.5	1.14–1.99	0.0041
							Basal: 0.69	0.5–0.94	0.019
NKLAM	2,765	mRNA	RNA-seq	NA	Low *vs*. High	DMFS	0.77	0.66–0.9	0.00086
PPIL2	2,765	mRNA	RNA-seq	Expressing difference	Low *vs*. High	DMFS	0.78	0.65–0.94	0.0085
Smurf1	2,765	mRNA	RNA-seq	NA	Low *vs*. High	DMFS	1.34	1.11–1.62	0.0022
Smurf2	2,765	mRNA	RNA-seq	NA	Low *vs*. High	DMFS	1.52	1.3–1.78	1.9e–07

IHC, immunohistochemical (IHC) staining; WB, western blot; OS, overall survival; DFS, disease-free survival; RFS, relapse-free survival; DMFS, distant metastasis-free survival; PPS, post-progression survival; BCSS, breast cancer–specific survival; HR, hazard ratio; 95%CI, 95% confidence intervals; RR, relative risk; NA, not available in the reference literature.

**Figure 4:**
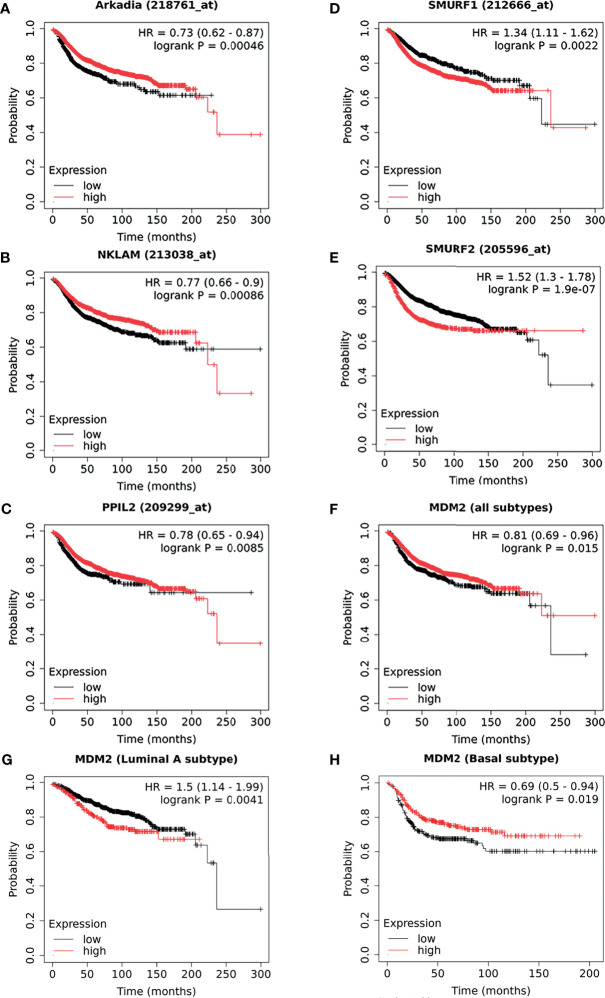
The survival curves comparing patients with high and low expressions of E3s in breast cancer patients. As a supplement to the literatures, the survival analysis of Arkadia **(A)**, NKLAM **(B)**, PPIL2 **(C)**, Smurf1 **(D)**, and Smurf2 **(E)**, which were illustrated as a Kaplan–Meier plot. Distant metastasis–free survival curves for high (red) and low (black) expression groups dichotomized at the optimal cut-point. The survival analysis of MDM2 in breast cancer was shown as all types **(F)**, luminal A **(G)** subtype, and basal subtype **(H)**, respectively. Logrank P-value and the hazard ratio with 95% confidence intervals was calculated. The threshold was adjusted to logrank P‐values at < 0.05.

By comparing with molecular mechanism, five E3 ligases have been found to be significantly correlated with the DMFS of breast cancer patients. As illustrated in Kaplan–Meier plots (**Figure 4**), we conducted the survival analysis of Arkadia (**Figure 4A**), NKLAM (**Figure 4B**), PPIL2 (**Figure 4C**), Smurf1 (**Figure 4D**), and Smurf2 (**Figure 4E**), which is consistent with the laboratory mechanism previously found. Patients with high expression of Arkadia (Hazard Ratio: 0.73; Logrank P: 0.00046), NKLAM (Hazard Ratio: 0.77; Logrank P: 0.00086), and PPIL2 (Hazard Ratio: 0.78; Logrank P: 0.0085), which have an inhibitory effect on metastasis, have higher survival probability. Patients with high expression of Smurf1 (Hazard Ratio: 1.34; Logrank P: 0.0022) and Smurf2 (Hazard Ratio: 1.52; Logrank P: 1.9e-07) have lower survival probability. Combining this analysis results with summarized literature reports, E3 ubiquitin ligases which are significantly correlated with patients’ survival were presented in **Table 2**.

Interestingly, we found that some results of survival analysis were contrary to the molecular mechanism. For example, if all subtypes of breast patients were considered together, a higher expression of MDM2 showed better survival (Hazard Ratio: 0.81; Logrank P: 0.015) (**Figure 4F**), which was obviously contrary to known molecular mechanisms. MDM2 has been well proven to be an E3 ubiquitin ligase that simultaneously targets tumor suppressor protein RB and P53 to degradation. We further analyzed different subtypes and found that it was opposite in patients with luminal A (Hazard Ratio: 1.5; Logrank P: 0.0041) (**Figure 4G**) and basal subtype (Hazard Ratio: 0.69; Logrank P: 0.019) (**Figure 4H**). This suggests that the heterogeneity of breast cancer cannot be ignored. In addition, it might be affected by the expression level or mutation of ubiquitin substrates, such as the status of p53 mutation or loss of RB. The context-dependent role of E3s and breast cancer subtypes need to be considered more in future survival analyses.

## 4 Discussion

In the past decades, dozens of studies have demonstrated that many E3 ubiquitin ligases play very important roles in breast cancer metastasis. E3 ligases were involved in the multiple steps of breast cancer metastasis, including EMT, invasion and migration, cell stemness, angiogenesis, and immunity response in the tumor microenvironment.

Typical E3s’ functions comprise of recognition and recruiting a specific protein to be modified and then catalyzing ubiquitin molecule discharge from an active-site cysteine onto the recruited substrate or a substrate-linked ubiquitin. Through this posttranslational modification, E3 ligases can alter the fate of their protein substrates, transducing different signals, which is critical for breast cancer metastasis. For example, the E3 ligase Cbl-b mediates the ubiquitination and degradation of EGFR, which inhibits metastatic breast cancer cells’ EMT. And another E3 ligase, MEX3C, catalyzes the tumor suppressor PTEN with K27-linked polyubiquitination and alters its enzymatic function, which leads to the accumulation of the master regulators of EMT, including twist family bHLH transcription factor 1 (TWIST1), Yes1-associated transcriptional regulator (YAP1), and SNAI1.

In light of the vital roles of E3 ubiquitin ligases in breast cancer metastasis, targeting them for cancer therapy has gained increasing attention. Notably, bortezomib, a proteasome inhibitor, was approved by the Food and Drug Administration (FDA) of United States to treat multiple myeloma and certain lymphomas, which encourages more and more researchers to screen the small molecular inhibitors of particular E3 ligases for anti-metastatic breast cancer. We queried with the key words of each E3 ubiquitin ligase names and their aliases in the AACT (Public Access to Aggregate Content of ClinicalTrials.gov, https://aact.ctti-clinicaltrials.org/) database, which is a publicly available relational database that contains information (protocol and result data elements) about every study registered in ClinicalTrials.gov. However, only the inhibitor for MDM2 has been registered for breast cancer treatment in undergoing clinical trials. It offers great opportunity for future pharmacological exploitation.

Though it is believed that inhibiting and redirecting ubiquitination *in vivo* are new therapeutic strategies, especially specific inhibitors of E3 ubiquitin ligase will be discovered and developed as a novel class of anticancer drugs in the foreseeable future, we are still facing significant challenges so far. Firstly, the specific substrate of E3 ligase had been elucidated in few studies and needs to be identified, especially in the process of breast cancer metastasis. Recent advances in high-throughput screening chemical methods have revolutionized our ability to match E3 ubiquitin ligases with their cellular targets (134), like the UBAIT (ubiquitin-activated interaction traps strategy), which relies on a ubiquitin molecule covalently fused to the E3 ligase of interest being charged onto E2 enzymes. Using the affinity enrichment of tagged UBAITs with following mass spectrometry can identify substrates of several E3s (135). Both *in vivo* and *in vitro* ubiquitin assays are also suggested to be used for the validation of E3 ligase substrates. Secondly, the substrate recognition specificity of E3 ligases needs to be understand more deeply in breast cancer metastasis, which is critical for the efficient small-molecule inhibition of substrate degradation. Comparing proteins modified in cell lysates *versus* when an E3 ligase is depleted will allow the identification of substrates (134, 136). Thirdly, structural bases and ubiquitin mechanisms facilitate with further exploit pharmacological strategy. For example, typical HECT family E3s harbor catalytic cysteines that first receive ubiquitin molecule from a bound E2~Ub intermediate and then directly deliver the ubiquitin to the substrate protein, which can pharmacologically target the catalytic cysteines of E3s (134). Instead, there are still several non-classical E3s whose structure and specific ubiquitin transfer mechanisms remain unknown. Finally, E3 ligases exhibit distinct or even opposite functions in different breast cancer subtypes, suggesting that a subtype-specific approach to E3 ligase substrates and inhibitor screening is required. A good example is K^+^ channel tetramerization domain 10 (KCTD10), a BTB protein, as an adaptor protein that forms E3 ubiquitin ligase complex with Cullin3. CUL3/KCTD10 ubiquitinated RhoB for K63-linked ubiquitin degradation and promote HER2-positive breast cancer cell proliferation (137, 138). Two downstream proteins of RhoB, RAC1 (Rho GTPase) and CNKSR1 (connector enhancer of kinase suppressor of Ras1), were found to be significantly correlated with the prognosis of HER2-positive breast cancer patients (138, 139). Beyond small-molecule inhibitors, proteolysis-targeting chimeras (PROTACs), which can induce the recruitment of E3 to target protein, have recently emerged as significant future therapeutic opportunities (140, 141).

In addition, breast cancer cell–secreted exosomes have been found to play roles in the microenvironment and enhance the invasiveness of recipient cells, which contribute to breast cancer invasion through the EGFR signaling (142, 143). An exosome-mediated delivery of the intrinsic PTEN-stabilizing factor PTEN-CT (Carbon Terminus) has been found to protect PTEN from E3 ligase–mediated proteasomal degradation and then inhibit breast cancer cell proliferation and migration (144). It suggests that not only intracellular ubiquitination but also intercellular ubiquitination (like exosome- mediated migratory delivery) should be followed with interest in the future.

With >700 E3 ligases in the human genome, including but not limited to the 54 E3s that have been identified to be involved in breast cancer metastasis, it has become clear that some of them are promising therapeutic targets or prognostic markers for breast cancer.

## Data Availability Statement

The original contributions presented in the study are included in the article/supplementary material. Further inquiries can be directed to the corresponding authors.

## Author Contributions

YW, JG and JL designed this study. YW contributed to the conception, acquisition, analysis, and interpretation of data and drafted the manuscript. JD and YZ contributed to the acquisition of data. Schematic diagrams were drawn by JD. JG and JL contributed to the interpretation of data and critically revised the manuscript. All authors contributed to the article and approved the submitted version.

## Funding

This work was supported by the National Natural Science Foundation of China (82003001, 82003227); China Postdoctoral Science Foundation (2019M663516,2021T140487); Post-Doctor Research Project, Sichuan University (20826041D4022); Post-Doctor Research Project, West China Hospital, Sichuan University (2018HXBH067); Sichuan Provincial Research Foundation for Basic Research (2020YFS0272); and Sichuan Science and Technology Program (2020YJ0046).

## Conflict of Interest

The authors declare that the research was conducted in the absence of any commercial or financial relationships that could be construed as a potential conflict of interest.

## Publisher’s Note

All claims expressed in this article are solely those of the authors and do not necessarily represent those of their affiliated organizations, or those of the publisher, the editors and the reviewers. Any product that may be evaluated in this article, or claim that may be made by its manufacturer, is not guaranteed or endorsed by the publisher.
